# Burkitt's lymphoma presenting with hypopituitarism: a case report and review of literature

**DOI:** 10.1530/EDM-14-0029

**Published:** 2014-07-01

**Authors:** Siew Hui Foo, Shahada A H Sobah

**Affiliations:** 1 Endocrine Unit, Department of MedicineSelayang HospitalSelangorMalaysia; 2 Department of HaematologyAmpang HospitalSelangorMalaysia

## Abstract

Hypopituitarism is a rare presentation of Burkitt's lymphoma (BL). The purpose of this report is to present a case of BL presenting with panhypopituitarism and to review other case reports of lymphoma presenting with pituitary dysfunction to highlight the distinguishing features of these cases from other benign aetiologies of pituitary dysfunction such as non-functioning pituitary adenomas. We reviewed a total of 11 cases of lymphoma presenting with pituitary dysfunction published from 1998 to 2013 including the present case. The demographics, clinical presentations, laboratory features, radiological findings, histological diagnosis, treatment administered and outcomes were described. Of the total number of patients, 45.5% of the cases had diffuse large B-cell lymphoma while 27.3% had BL. Anterior pituitary dysfunction was more common than posterior pituitary dysfunction at presentation. The other common associated presenting symptoms were painful ophthalmoplegia, cranial nerve palsies and constitutional symptoms. Hypothalamic–pituitary abnormalities were often demonstrated radiologically to be associated with cavernous sinus and/or stalk involvement. All patients who completed immunochemotherapy responded haematologically. Pituitary dysfunction also improved in most cases although the recovery tended to be partial. In conclusion, a high index of suspicion of underlying malignancy, such as lymphoma, should be present in patients presenting with acute pituitary dysfunction associated with painful ophthalmoplegia, rapidly evolving neurological features, radiological features atypical of a pituitary adenoma and constitutional symptoms. An early diagnosis is essential as prompt initiation of definitive therapy will induce disease remission and recovery of pituitary dysfunction.

**Learning points:**

- Hypopituitarism may be the presenting symptom of lymphoma in the absence of associated overt symptoms or signs of a haematological malignancy resulting in delay in diagnosis and institution of treatment.
- Pituitary dysfunction due to tumour infiltration has a greater tendency to involve the posterior pituitary and infundibulum resulting in diabetes insipidus and hyperprolactinaemia compared with a non-functioning pituitary adenoma.
- The common associated symptoms of hypopituitarism due to lymphoma infiltration of the hypothalamic–pituitary system include painful ophthalmoplegia, cranial nerve palsies and constitutional symptoms.
- Radiological abnormalities of the hypothalamic–pituitary region are usually present and often associated with cavernous sinus or stalk involvement.
- With early institution of definitive treatment, both haematological response and improvement of pituitary dysfunction are expected although the reversal of hypopituitarism tends to be partial and delayed.
- A high index of suspicion of underlying malignancy such as lymphoma should be present in patients presenting with acute pituitary dysfunction associated with painful ophthalmoplegia, radiological features atypical of pituitary adenomas and constitutional symptoms to enable early diagnosis and prompt initiation of definitive therapy.

## Background

Hypopituitarism is a rare presentation of Burkitt's lymphoma (BL), a highly aggressive type of B-cell non-Hodgkin's lymphoma (NHL) associated with rapid and aggressive clinical course (1). The diagnosis of BL is considered a medical emergency and requires immediate constitution of definitive therapy. BL commonly presents with jaw or facial bone involvement, and hypopituitarism at presentation is rare and may lead to a delay in diagnosis especially if not associated with overt signs and symptoms of systemic lymphoma. The purpose of this paper is to present a rare case of BL presenting with panhypopituitarism from our institution and to review other case reports or series of lymphoma presenting with pituitary dysfunction to highlight the distinguishing features of these cases from other common or benign aetiologies of pituitary dysfunction such as non-functioning pituitary adenomas.

## Case presentation, management and outcome

A 39-year-old Malay woman presented at our institution in July 2013 with a 1-week history of painful diplopia associated with drooping of right upper eyelid preceded by a 2-week history of intermittent pre-syncopal attack and a 5-day history of vomiting. She had no previous medical illness. There was a 1-month history of weight loss and anorexia associated with occasional headache. Her menstruation had been regular up to 1-month before presentation. She also reported intermittent constipation associated with dry skin recently. Socially, she was married with four children. Her last child was born in 2004. There was no history of *post partum* haemorrhage or traumatic brain injury.

On examination, the patient's blood pressure was 111/70 mmHg with a pulse rate of 105 beats/min. There was a complete right third cranial nerve palsy along with the right fourth and sixth cranial nerve palsies. Respiratory and abdominal systems were unremarkable. The capillary blood glucose was 4.9 mmol/l. There was no visual field defect on objective assessment later.

A contrasted computed tomography (CT) brain performed revealed a widened sella turcica with an enhancing mass lesion measuring 1.6×1.4 cm associated with symmetrical enhancement of both cavernous sinuses. Laboratory investigations revealed evidence of panhypopituitarism associated with hyperprolactinaemia, raised lactate dehydrogenase (LDH), mild renal impairment, hyponatraemia and hypokalaemia (Table 1). The provisional diagnosis was pituitary macroadenoma associated with panhypopituitarism and mass effect on the right cavernous sinus.

**Table:** Laboratory investigation results upon presentation (July 2013) and with disease progression (August 2013)

**Parameters**	**July 2013**	**August 2013**	**Reference range**
Haematology			
Total white (×10^3^/μl)	7.6	7.6	4–11
Haemoglobin (g/dl)	12.4	**7.5**	11.5–16.5
Platelet (×10^3^/μl)	157	**62**	150–400
Biochemistry			
Urea (mmol/l)	3.1	**12.5**	1.7–8.3
Sodium (mmol/l)	**131**	**130**	135–150
Potassium (mmol/l)	**3.3**	4.0	3.5–5.0
Creatinine (μmol/l)	**112**	**98**	44–88
Total protein (g/dl)	77	70	66–87
Albumin (g/dl)	43	36	35–50
Bilirubin (μmol/l)	18	16	≤20
ALP (IU/l)	101	116	53–141
ALT (IU/l)	**36**	**51**	≤33
AST (IU/l)	**92**	**98**	≤31
LDH (IU/l)	**1368**	**2130**	140–271
Creatinine kinase (U/l)	**282**	69	25–170
Hormonal profiles			
0800 h cortisol (nmol/l)	**45**		119–618
ACTH (pmol/l)	**4**		0–10
Free thyroxine (pmol/l)	**9.0**		11.8–23.2
TSH (mU/l)	**0.38**		0.4–5.5
FSH (IU/l)	2.0		0.8–7.5 (luteal)
LH (IU/l)	**0.4**		0.8–27.1 (luteal)
Oestradiol (pmol/l)	**<44**		550–845
Prolactin (mU/l)	**2968**		59–169
IGF1 (μg/l)	25		22–197 (age 31–40)

Bold values are out of the reference range. ALP, alkaline phosphatase; ALT, alanine transferase; AST, aspartate transferase; LDH, lactate dehydrogenase; ACTH, adrenocorticotropic hormone; TSH, thyroid-stimulating hormone; FSH, follicle-stimulating hormone; LH, luteinising hormone; IGF1, insulin-like growth factor 1.

**Figure 1:**
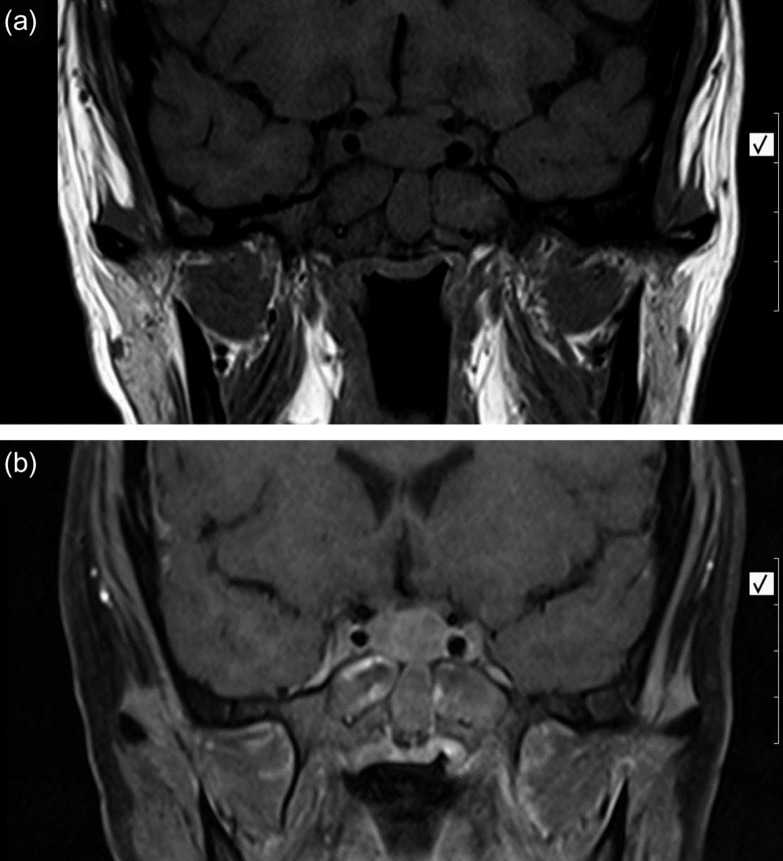
(a) A T1-weighted magnetic resonance imaging (MRI) of the sellar region showing a diffusely enlarged pituitary gland measuring 1.6 (W)×1.1 (H) cm with lateral extensions encasing both cavernous sinuses bilaterally and mild compression of the optic chiasm. (b) A T1-weighted MRI of the sellar region after contrast showing heterogeneous enhancement of the enlarged pituitary gland along with the thickened lateral walls of both cavernous sinuses.

Hydrocortisone and l-thyroxine replacement was initiated. While waiting for magnetic resonance imaging (MRI) of the pituitary, the patient developed nosocomial pneumonia requiring intensive care. She was covered with broad-spectrum antibiotics and stress dose of glucocorticoids. The MRI of the pituitary reported a diffusely enlarged pituitary gland with heterogeneous enhancement, mild optic chiasm compression and lateral extension encasing both cavernous internal carotid arteries bilaterally. The lateral walls of both cavernous sinuses were diffusely thickened with contrast enhancement. The radiological impression was pituitary macroadenoma. The differential diagnosis was inflammatory or infiltrative disorders of the pituitary gland (Fig. 1).

**Figure 2:**
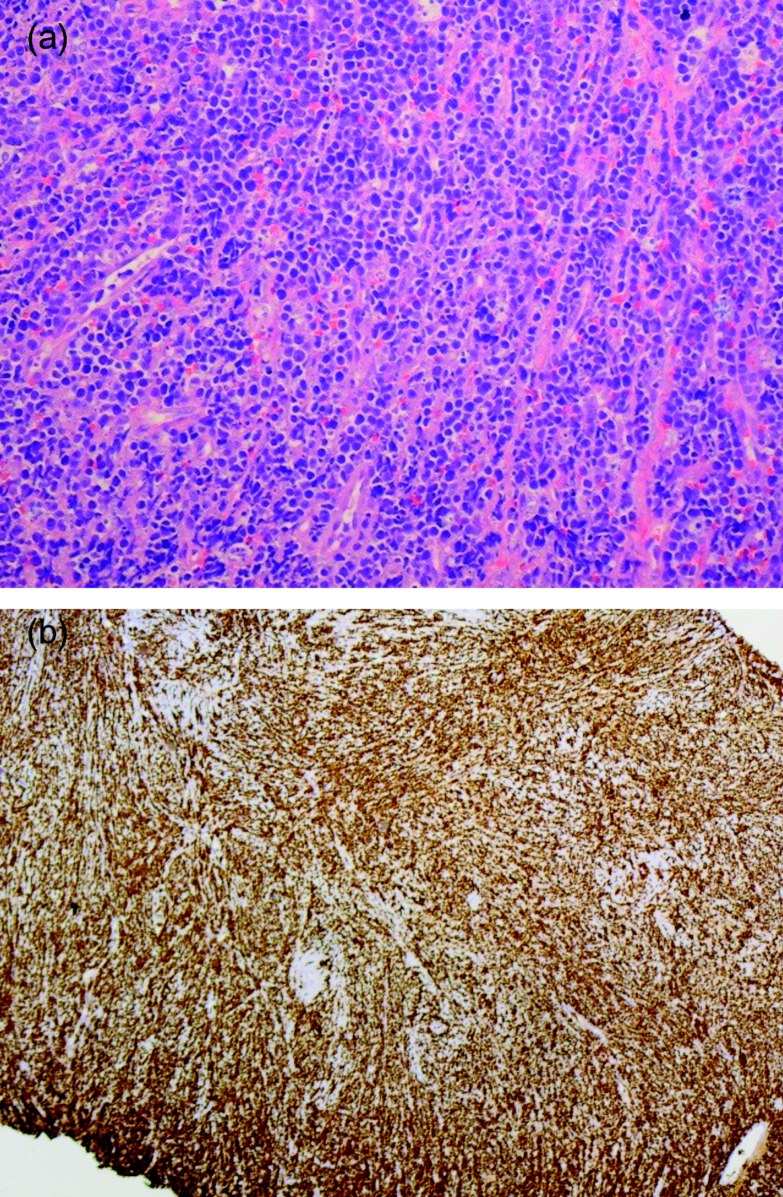
(a) Haematoxylin and eosin stain (20× original magnification). Sheets of monomorphic neoplastic lymphoid cells with pleomorphic nuclei, coarse chromatin and little cytoplasm. (b) Ki-67 stain (4× original magnification). Malignant lymphoid cells stained heavily with a Ki-67 index of 100%.

The patient subsequently complained of swellings in her breasts and over the left jaw. Physical examination revealed new findings of left lower motor neuron palsy of the seventh cranial nerve associated with proptosis of both eyes, multiple tender breast lumps over all quadrants bilaterally and a 2×3 cm exophytic lesion that was firm with irregular surface over the posterior left buccal mucosa. There was no palpable cervical or peripheral lymphadenopathy. Repeated laboratory investigations revealed the presence of anaemia and thrombocytopaenia (Table 1). The peripheral blood film showed moderate normocytic normochromic anaemia with thrombocytopaenia. A mammogram performed revealed the presence of large nodular lesions bilaterally with Breast Imaging Reporting and Data System (BI-RADS) type 4 density highly suspicious of malignancy. Biopsy was performed for both the buccal and breast lesions. The preliminary histopathological examination of the buccal biopsy showed tissue infiltration by sheets of neoplastic cells with pleomorphic nuclei, coarse chromatin and little cytoplasm. Numerous mitotic figures were observed. The histological diagnosis was malignant B-cell lymphoma (Fig. 2a). Similar findings were reported in the breast biopsy.

Based on the compilations of signs and symptoms in the patient, the diagnosis was revised to left buccal B-cell lymphoma with progressive local, regional and systemic infiltration including the left seventh cranial nerve, retro-orbital tissue bilaterally, anterior pituitary and stalk along with both cavernous sinuses, breasts and bone marrow. The patient was taken over by a haematologist from another institution for definitive therapy in August 2013. A CT of the neck, thorax and abdomen revealed extensive intra-abdominal para-aortic lymphadenopathy with bilateral breast masses. Further immunohistochemical staining indicated that the malignant lymphoid cells were positive for CD20 and CD10 and negative for BCL2; Ki-67 was 100% (Fig. 2b). The final diagnosis, based on the aggressive clinical progression, morphological appearance, immunohistochemical staining pattern and a high proliferative index, was revised to stage IV BL of the jaw with local and systemic infiltration of the pituitary, breasts and bone marrow. Unfortunately, the patient developed neutropenic sepsis during the course of high-dose methotrexate-based chemotherapy and subsequently succumbed to the complications of chemotherapy in September 2013.

## Discussion

The most common cause of hypopituitarism associated with a sellar mass is non-functioning pituitary adenoma. Pituitary metastases constitute only 1–5% of all sellar tumours, commonly due to malignancy from the lung in men or breast in women (2). The incidence of lymphoma as an aetiology of pituitary tumour metastases is only 0.5% (3). Most of these cases have lymphoma manifestations preceding pituitary dysfunction. Evidence of pituitary dysfunction associated with a sellar mass at the onset is extremely rare. This report describes a rare case of BL presenting with hypopituitarism. We also have reviewed ten other cases of lymphoma presenting with pituitary dysfunction as reported in the literature from 1998 to 2013. The individual patient demographics, clinical presentations, laboratory features, radiological findings, histological diagnosis, treatment administered and outcomes were summarised in Table 2 including those of the present case. There were six males and five females aged 19–70 years (mean, 51.9±19.0 years). Five cases (45.5%) were diffuse large B-cell lymphoma (DLBCL) or its subtype of intravascular large B-cell lymphoma, 3 (27.3%) were BL, two were non-specific B-cell NHL, while one was not specified. DLBCL was the most common subtype reported followed by BL. Although DLBCL is also the most common subtype of NHL reported in the literature at 35% as well as the most common type of lymphoma involving the hypothalamic–pituitary system, BL is rare (1) (3) (4). It constitutes only 1% of all the NHL in contrast to 27.3% in this series (1). This implies that among the different subtypes of lymphoma, BL may be more likely than other subtypes to involve the pituitary right from the disease onset.

**Table:** Demographics, clinical features, treatment and outcomes of 11 patients with lymphoma presenting with pituitary dysfunction

**Reference**	**Age** (years)	**Gender**	**Endocrinopathies**	**Other clinical features**	**Radiological findings**	**Histological diagnosis**	**Treatment and outcome**
PC	39	Female	Panhypopituitarism with low cortisol, FSH/LH/oestradiol, FT_4_/TSH; raised prolactin	Painful ophthalmoplegia, headache, weight loss, anorexia and raised LDH	Pituitary MRI: diffusely enlarged pituitary gland with thickened walls of cavernous sinus	Burkitt's lymphoma (buccal biopsy)	High-dose methotrexate-based chemotherapy, died of neutropenic sepsis 2 months after initial presentation
(4)	59	Male	Panhypopituitarism with low cortisol, testosterone, FT_4_; raised prolactin; DI	Partial left third CN palsy, headache and raised ESR/CRP	Pituitary MRI: diffusely enlarged pituitary gland with cavernous sinus encasement and meningeal enhancement	Diffuse large B-cell lymphoma (adrenal biopsy)	NR
(9)	67	Female	Panhypopituitarism	Malaise, weight loss, fever, anaemia, thrombocytopaenia and raised LDH/ESR	Pituitary MRI: partially empty sella	Intravascular large B-cell lymphoma (bone marrow biopsy)	R-CHOP received. haematological remission achieved after 18 months. Complete reversal of hypopituitarism achieved after 2 years
(5)	70	Female	Partial hypopituitarism with low FSH/LH, raised prolactin; DI	Right third CN palsy, fatigue and thrombocytopaenia	Pituitary MRI: sellar mass involving the pituitary gland and stalk with extension into right cavernous and sphenoid sinus	High-grade B-cell NHL (pituitary gland histology)	Transsphenoidal pituitary surgery. Died 1 week post-operatively
(8)	63	Male	Panhypopituitarism	Left fifth CN palsy, anorexia and night sweats	Pituitary MRI: hypothalamic mass with stalk extension	Diffuse large B-cell lymphoma (liver biopsy)	Remission achieved after several cycles of CNS-targeted chemotherapy. PET–CT negative but hypopituitarism persisted
(10)	65	Female	Panhypopituitarism with low ACTH/cortisol, FSH/LH, FT_4_/TSH and IGF1	Lethargy, anorexia, pedal oedema and pancytopenia	Pituitary MRI: normal PET: metabolically active areas in the pituitary, neck, axilla and liver CT abdomen: intra-abdominal lymphadenopathy	Diffuse large B-cell lymphoma (liver biopsy)	Haematological remission with reversal of hypopituitarism achieved at 10 months after completion of R-CHOP
(11)	19	Female	Panhypopituitarism with low cortisol, FSH/LH and FT_4_/TSH	Right third CN palsy with proptosis, left breast mass and fatigue	Pituitary MRI: a 2.9×2.5 cm parasellar mass involving the right cavernous sinus with suprasellar extension	Burkitt's lymphoma (breast biopsy)	Intrathecal CODOX-M-IVAC resulted in resolution of pituitary mass with partial reversal of panhypopituitarism
(3)	26	Male	DI	Anaemia; raised LDH	Pituitary MRI: small hypophyseal fossa with absence of high signals from posterior pituitary with thickened stalk CT abdomen: bilaterally enlarged kidneys	Burkitt's lymphoma (renal biopsy)	Partial haematological remission achieved after four cycles of EPOCH followed by two cycles of mitoxantrone, cytarabine, dexamethasone and thalidomide
(12)	63	Male	Panhypopituitarism with low ACTH/cortisol, FT_4_/TSH testosterone/DHEA/aldosterone; DI	Left ptosis, miosis, hypohidrosis, anaemia; raised LDH	Pituitary MRI: a 13 mm suprasellar lesion with thickened stalk ^18^F-FDG PET–CT: high uptake in the pituitary gland, bilateral adrenal glands and apex of the left lung	Diffuse large B-cell lymphoma (pituitary biopsy)	Six cycles of R-CHOP followed by two cycles of high-dose methotrexate-based chemotherapy and autologous haemopoietic stem cell transplant. Complete resolution of all radiological lesions with partial remission of hypopituitarism
(6)	68	Male	Panhypopituitarism and DI	Generalised lymphadenopathy	MRI: thickened pituitary stalk	Lymphoma (axillary lymph node biopsy)	Resolution of thickened pituitary stalk but persistence of hypopituitarism after chemotherapy
(13)	32	Male	DI	Sixth CN palsy, raised ESR	Gallium-68 scan: uptake in iliac bone and skull with extension into right cavernous sinus	B-cell lymphoma (bone marrow biopsy)	Chemotherapy followed by autologous peripheral stem cell transplant and local radiotherapy. Lymphoma symptoms resolved while DI improved

PC, present case; FSH, follicle-stimulating hormone; LH, luteinising hormone; FT_4_, free thyroxine; TSH, thyroid-stimulating hormone; LDH, lactate dehydrogenase; MRI, magnetic resonance imaging; DI, diabetes insipidus; CN, cranial nerve; ESR, erythrocyte sedimentation rate; CRP, C-reactive protein; NR, not reported; R-CHOP, rituximab, cyclophosphamide, doxorubicin, vincristine and prednisone; NHL, non-Hodgkin's lymphoma; PET, positron emission tomography; CT, computed tomography; ACTH, adrenocorticotropic hormone; IGF1, insulin-like growth factor; CODOX-M-IVAC, cyclophosphamide, vincristine, doxorubicin, high-dose methotrexate, ifosfamide, etoposide and high-dose cytarabine; EPOCH, cyclophosphamide, epirubicin, vincristine, etoposide and prednisone; ^18^F-FDG, ^18^F-fluorodeoxyglucose.

The most common clinical presentation in this case series was pan or partial anterior hypopituitarism at 81.8% followed by diabetes insipidus, ophthalmoplegia and constitutional symptoms at 54.5% each. The ophthalmoplegia was typically painful, mostly due to third cranial nerve palsies and occasionally associated with fourth, fifth, sixth or seventh cranial nerve palsies as well. This is in contrast to pituitary adenomas, with the most common neurological manifestation as a visual field defect due to compression of the optic chiasm. Yang *et al*. reviewed 19 cases of NHL associated with pituitary metastases and found that posterior pituitary dysfunction was more common than anterior pituitary dysfunction at 73.7 vs 42.1% respectively. However, majority (68.4%) of the cases presented with lymphoma manifestations and only exhibited pituitary dysfunction later in the course of the disease. Among the four patients who had endocrine manifestations first, the lymphoma manifestations were delayed between 6 weeks and 3 years later (3).

Of the nine patients with laboratory evidence of anterior pituitary dysfunction in this series, 8 (88.9%) had panhypopituitarism involving the gonadotrophin, adrenocorticotrophic hormone (ACTH)–cortisol and thyrotrophin axis while one patient had isolated hypogonadotrophic hypogonadism. Although the low gonadotrophin levels in the last patient were deemed to be due to direct infiltration of the anterior pituitary by the author, this may, however, could also be the result of suppression by hyperprolactinaemia (5). Four of those nine patients with anterior pituitary dysfunction also had concurrent posterior pituitary dysfunction manifested as diabetes insipidus while two other patients presented with isolated diabetes insipidus. This is in comparison to non-functioning pituitary adenomas, which rarely have posterior pituitary involvement. Diabetes insipidus is reported only in 1% of pituitary adenomas (5). Mild to moderate hyperprolactinaemia reported in the three patients (27.3%) in this series was most probably secondary to pituitary stalk involvement compromising dopamine inhibition of prolactin production. Pituitary metastases have a greater tendency to involve the posterior pituitary and infundibulum resulting in diabetes insipidus and mild to moderate hyperprolactinaemia. This is due to the direct blood supply by the hypophyseal arteries from the systemic circulation (2) (5) (6). Common laboratory features of lymphoma such as haematological abnormalities, raised LDH or inflammatory markers were only reported in 3 (27.3%), 4 (36.4%) and 3 (27.3%) patients respectively in this series.

Radiological evidence of hypothalamic–pituitary abnormalities were demonstrated in ten out of 11 patients with eight of them presenting with sellar, parasellar or hypothalamic mass; one was described as thickened pituitary stalk while the other was reported as partially empty sella. A high proportion of patients (81.8%) had evidence of cavernous sinus or stalk involvement. By contrast, only 9.6% of pituitary adenomas had evidence of cavernous sinus involvement while stalk involvement is highly unusual (7). Suprasellar extension is a relatively common finding in non-functioning pituitary macroadenomas. This is, however, uncommon in this case series. The presence of dural enhancement has also been described as a distinguishing feature of pituitary lymphoma, which is usually absent in pituitary adenomas (5) (8). It is difficult to distinguish pituitary metastases due to lymphoma from other malignant tumours radiologically unless a pituitary biopsy is performed. However, even an image-guided biopsy is associated with a significant risk of injury to the surrounding critical neurovascular structures and is often not performed. The diagnosis is best made in correlation with the overall clinical and laboratory features.

Treatment and outcome were reported for the ten patients from the literature. All were treated with intensive chemotherapy with or without immunotherapy and they survived except for two cases. One died after transsphenoidal surgery of the pituitary while the present case died of neutropenic sepsis during the course of chemotherapy. All the eight patients who managed to complete their chemotherapy responded haematologically. Complete or partial remission of pituitary dysfunction occurred in six patients (75%). The limited duration of follow-up in most cases may be the limiting factor here as complete restoration of hypopituitarism may take more than 2 years (9). The radiological outcome may be incongruent with the biochemical outcome as three patients were documented to achieve complete radiological resolution although the hypopituitarism persisted or only partially recovered. This highlights the importance of a repeat hormonal assessment on top of radiological assessment in the evaluation of pituitary function after chemotherapy.

In conclusion, hypopituitarism is a rare presentation of BL. It is difficult to differentiate pituitary infiltration by lymphoma from pituitary adenomas or other sellar tumours. This is only the third case report of BL presenting with hypopituitarism. A high index of suspicion of underlying malignancy, especially lymphoma, should be raised in patients presenting with acute pituitary dysfunction especially when associated with diabetes insipidus, painful ophthalmoplegia, rapidly evolving neurological features due to local infiltration, constitutional symptoms and radiological features atypical of a pituitary adenoma with cavernous sinus or stalk involvement. An early diagnosis is essential especially for aggressive subtypes of lymphoma such as BL as prompt institution of effective therapy will have a high probability to induce remission and recovery of pituitary dysfunction to avoid premature mortality or morbidity.

## Patient consent

A written informed consent was not obtained from the patient as the patient is dead. The patient's identity remained anonymous in this report.

## Author contribution statement

S H Foo was involved in the patient care as the endocrinologist in charge, prepared the manuscript, and reviewed the literature. S A H Sobah prepared the histological pictographs and was the haematologist in charge after the patient was taken over to another institution.
